# How Were Healthcare Workers after Anti-SARS-CoV-2 Vaccination? A Study of the Emotional Side Effects of Vaccination

**DOI:** 10.3390/vaccines10060854

**Published:** 2022-05-26

**Authors:** Maria Grazia Mada Logrieco, Giuseppe Logrieco, Ilaria Nicolì, Francesca Pignatelli, Francesca Lionetti, Francesco Traglia, Maria Spinelli, Alberto Di Domenico, Mirco Fasolo

**Affiliations:** 1 Department of Neuroscience, Imaging and Clinical Sciences, University “G. d’Annunzio” Chieti-Pescara, Via dei Vestini 33, 66100 Chieti, Italy; ilaria.nicoli@unich.it (I.N.); francesca.lionetti@unich.it (F.L.); maria.spinelli@unich.it (M.S.); alberto.didomenico@unich.it (A.D.D.); mirco.fasolo@unich.it (M.F.); 2 Residency School of Pediatrics, University of Rome “Tor Vergata”, Via Montpellier 1, 00133 Rome, Italy; giuseppe.logrieco@opbg.net (G.L.); francesca.pignatelli@opbg.net (F.P.); 3 Department of Biomedicine and Prevention, University of Rome “Tor Vergata”, Via Montpellier 1, 00133 Rome, Italy; francesco.traglia@alumni.uniroma2.eu

**Keywords:** anti-SARS-CoV-2 vaccine, healthcare workers, emotions, vaccine uptake, information, vaccine promotion

## Abstract

Anti-SARS-CoV-2 vaccines appear to be the only escape from the COVID-19 pandemic. As healthcare workers were among the first in society to be vaccinated, understanding their emotional status post-vaccination is fundamental to the promotion of COVID-19 vaccines among the rest of society. The aims of this study were to investigate the predictors of positive and negative emotions experienced by healthcare workers after being vaccinated and to understand whether those emotions were related to the modalities of vaccine promotion within the community. A cohort of 5790 Italian healthcare workers completed an original online survey regarding their experience with anti-SARS-CoV-2 vaccines and reported on a series of personal and environmental factors. The data obtained show that increased risk perception of COVID-19, vaccine confidence and receipt of greater quantities of information regarding vaccines are predictors of a more positive emotional state post-vaccination. Predictors of a more negative emotional state are older age, lower education, lower confidence and receipt of smaller quantities of information, in addition to neurotic personality traits and high risk perception of COVID-19. Importantly, vaccination promotion may be favoured by a happy emotional status after vaccination. This study can serve as a source of guidelines for the promotion of COVID-19 vaccination among healthcare workers and laypeople.

## 1. Introduction

Coronavirus disease 2019 (COVID-19) has spread quickly all over the world [1,2]. In the absence of an effective therapy or vaccine, governments mandated restrictive measures to slow the spread of the virus and manage healthcare service provision [2,3]. Italy was the first country after China to impose restrictive measures on 9 March 2020. This precipitated the entire population into a state of high psychological distress. Many studies (see, for example, [4,5,6]) documented the psychological impact of lockdown. The need for an approved vaccine to protect populations from the virus was compelling [7,8,9]. On 9 November 2020, the candidate vaccine against COVID-19 announced by Pfizer and BioNTech achieved success in the first interim analysis. Italy was the third European country to begin an anti-SARS-CoV-2 vaccination campaign [10]. The vaccination campaign was initially aimed at healthcare professionals and other categories of frontline workers in order to mitigate the high daily risk of contagion within this population [11]. Subsequently, voluntary vaccination was offered to the rest of the population and an information campaign to promote vaccine uptake began. It was reported in the literature that people trusted COVID-19 information most when it came from healthcare professionals and health officials [12]. Public health messaging is fundamental to countering misinformation and improving trust in anti-SARS-CoV-2 vaccines. In fact, immunization programs are only successful when there are high rates of coverage [12]. To achieve this, it might be critical to understand the emotional impact of the jumpstart phase of the vaccination campaign for the Italian population. What may be the factors which predict a better emotional impact of the vaccine? Does a positive post-vaccination emotional impact influence how vaccinated people promote the acceptance, uptake and diffusion of anti-SARS-CoV-2 vaccines within their community? Considering the uniqueness of the case and future mass immunization, an informative approach may be needed to identify the psychological factors that increase the positive emotional impact of vaccination and promote attitudes conducive to vaccine acceptance and uptake. The present study aimed to contribute to this area of research by examining several potential predictors of an increase in the positive emotional impact of the vaccine during the first wave of vaccination (from January to March 2021) and the effect of such an impact on the vaccine promotion campaign. This is necessary for the tailoring of public health messages to increase vaccine uptake and awareness. In 2015, the World Health Organization Strategic Advisory Group of Experts on Immunization defined vaccine hesitancy as a delay in acceptance or refusal of vaccination despite the availability of vaccination services [13,14,15,16]. Concern about vaccine hesitancy is growing worldwide [17]; in fact, WHO (2019) [18] identified it as one of the top ten global health threats in 2019. Many factors might be involved in the process, all affecting individual predispositions toward vaccine uptake and consequent emotional reactions after being vaccinated. We aimed to explore both personal and environmental factors: sociodemographic characteristics, personality traits, vaccine confidence, COVID-19 perceived risk and the quality and quantity of information received regarding anti-SARS-CoV-2 vaccination.

### 1.1. The Personal and Environmental Factors Considered

The first set of factors considered were sociodemographic characteristics. The lack of success of mass immunization programs is often related to demographic factors [19,20,21]. Previous studies showed that those who were resistant to vaccines were more likely to be younger [3,6,21], have a lower education [12,21,22,23] and be male [3,6,21]. Taking these findings into account, it appeared to be essential to investigate whether these factors had an impact on emotional status after vaccination. The second set of factors considered were personality traits. The literature has shown that there is a strong relationship between personality traits and emotions. For example, McCrae and Costa 1991 [24] demonstrated that extraversive traits correlate with positive affect and that neurotic traits correlate with negative affect [25]. Considering the strong relationship between personality traits and emotions [26,27], we investigated personality characteristics as factors that could have an impact on emotional status after vaccination. The third factor considered was vaccine confidence. Due to the urgent need to combat COVID-19, vaccines of diverse types were developed [28]. This led to widespread public confusion regarding the different types of vaccines and their related side effects [29,30], encouraging parts of the population to distrust some kinds of anti-SARS-CoV-2 vaccine in particular and consequently leading to many people’s refusal to get vaccinated. From a psychological perspective, an important correlate of vaccination behavior is vaccine confidence, which involves attitudes and beliefs related to the benefits and safety of vaccines, as well as trust in vaccine providers [31,32,33,34,35]. Confidence in the benefits and safety of vaccines was highest among doctors and increased with level of education. Furthermore, the literature suggests that high confidence among healthcare workers is important in maintaining high vaccine uptake in the general population [36]. Thus, considering that the general population appears to view even approved COVID-19 vaccines as “experimental” given their novelty [37], we still do not know whether vaccine confidence has an impact on emotional status after vaccination. The fourth factor considered was COVID-19 perceived risk. The COVID-19 pandemic has shown higher severity in terms of transmissibility and mortality compared to past pandemics of influenza [38]. This plight was even more challenging for healthcare professionals [39,40]. From an analysis of the literature, perceived risk is a fundamental factor in vaccination acceptance [12,35] but no studies to date have considered whether this factor has an impact on emotional status after vaccination. The fifth factor considered was information received before vaccination. In many countries, vaccine misinformation presents substantial obstacles to achieving mass immunity [41,42] and it strongly affected acceptance and uptake of anti-SARS-CoV-2 vaccines [3,6,18,43,44,45]. Given the spread of severe and frightening news stories about vaccines, the way in which risks are communicated to the public is crucial to maintaining trust [27]. Indeed, “infodemiology” surveillance has been deemed a necessity [46,47,48]. Considering the huge amount of information that bombards us in our daily lives, the importance of understanding whether the quality and the amount of information regarding anti-SARS-CoV-2 vaccines had an impact on the emotional status of healthcare workers after vaccination emerged as a study aim. The sixth factor considered was vaccine promotion. Responsibility for public health messages and vaccine promotion primarily lies with governments, scientists and healthcare professionals [3]. Findings regarding acceptance or refusal of vaccines [3,21] and reports of trial participants’ lived experiences [49] underline the need for vaccination promotion campaigns [3,21,49,50]. Moreover, medical recommendation of vaccination is consistently shown to be one of the most important factors driving vaccination intention formation [36,51,52,53], and healthcare workers are considered to be the most reliable sources of information regarding vaccines [54,55,56,57,58]. A lack of recommendation is mentioned as a reason for non-vaccination [54,59,60,61]. Hence, it is necessary to investigate the modalities of vaccine promotion by healthcare providers after anti-SARS-CoV-2 vaccination came on stream.

### 1.2. The Present Study

The COVID-19 pandemic has had an enormous impact on healthcare systems which have had to deal with an unexpected increase in workloads in a context of uncertainty and powerlessness, with healthcare providers more vulnerable to infection due to direct contact with patients, which, in turn, increased their concerns about infecting their families and colleagues [39,40]. Indeed, this population has experienced high levels of burnout and psychological symptoms [62]. Considering that healthcare workers were the first to be vaccinated and that their messaging about vaccination had an important impact on the acceptance or refusal of vaccination [36,51,52,53], we wanted to consider the factors that contributed to a positive or negative emotional impact after receiving a vaccine and how the emotional impact of vaccination is related to the modalities of promotion of vaccine uptake within a community. Indeed, in contrast to other studies, our study aimed to evaluate the emotional status of healthcare workers after vaccination and link these statuses to the modalities of vaccine promotion within the relevant communities. Such an investigation is necessary in order to reach the highest possible level of vaccination coverage [63,64,65]. To our knowledge, this is the first study to approach this subject that includes reference to a vaccinated population.

## 2. Methods

### 2.1. Participants

The survey population was composed of 5790 Italian healthcare workers who received an anti-SARS-CoV-2 vaccine. Among them, 4775 were females (age range: 24–33, *n* = 1877, 39.3%; age range: 34–49, *n* = 2050, 42.9%; age range: 50–65, *n* = 809, 16.9%; age range: 66–80, *n* = 35, 0.7%; age range: 81–96, *n* = 1, 0.1%) and 990 males (age range: 24–33, *n* = 430, 43.4%; age range: 34–49, *n* = 323, 32.6%; age range: 50–65, *n* = 208, 21%; age range: 66–80, *n* = 26, 2.6%; age range: 81–96, *n* = 3, 0.3%).

The sample was distributed throughout the Italian peninsula as follows: 1451 participants were living in the South of Italy (24.4%) at the time of sampling, 1997 in the Central area (34.5%) and 2341 in the North (40.2%) [66]. Twenty-five (0.4%) participants had a middle school education, 323 (5.6%) had a high school education, 1179 (20.4%) had a bachelor’s degree, 1820 (31.4%) had a master’s degree and 2402 (41.5%) had a postgraduate formation. The sample contained 3,211 (55.5%) medical doctors (26.8%), nurses and other healthcare figures (Table A1). (Italian medical doctor reference population: 404,000; Italian nurse reference population: 360,000).

### 2.2. Procedure

Participants involved in the study filled out an anonymous online Qualtrics Survey (2020) [67] after reading the written consent form and explicitly agreeing to take part in the study voluntarily. The survey was shared via social media (Facebook and WhatsApp) for a limited time, from 26 January to 10 March 2021, including the first phase of vaccination for healthcare workers. There was no monetary compensation for participating. The study was approved by the ethical committee of the Department of Neuroscience Imaging and Clinical Science of the University of Chieti-Pescara (ethical approval number: DNISC richvy4ur 1659) and was conducted according to American Psychological Association guidelines in accordance with the 1975 Helsinki Declaration.

### 2.3. Materials

For the purpose of the study, the survey was reviewed and edited by a team of expert psychologists, medical doctors and statisticians (16, 3). The survey consisted of three parts preceded by a short introduction:

#### 2.3.1. Sociodemographic Characteristics

The first set of factors investigated were the sociodemographic characteristics of the participants, including age, sex, school education and region of residence.

#### 2.3.2. Your Experience with Anti-SARS-CoV-2 Vaccine

The second area was an original survey named “Your experience with anti-SARS-CoV-2 vaccine” and consisted of seven parts:

The first part concerned the healthcare worker’s type of job qualification. We also asked whether they were working in the COVID-19 department of a hospital.

The second part investigated the eventual chronic pathologies of the subject.

The third part concerned COVID-19. In this section, we asked participants how high their perceived risk of COVID-19 was according to a Likert scale (1 = not high at all; 7 = very high), whether they had had COVID and, if so, whether they had experienced collateral symptoms, as well as the duration of their quarantine.

The fourth part was about the vaccine. We asked participants to rate their vaccination confidence (with respect to vaccination effectiveness) on a Likert scale (1 = not confident at all; 7 = very confident). Participants were also asked whether they had any kind of allergic symptoms after other vaccinations.

We then focused our questions on anti-SARS-CoV-2 vaccines. Specifically, we asked which anti-SARS-CoV-2 vaccine they had received and when, and about any possible collateral symptoms they experienced after the vaccination.

For those who had only received their first injection of an anti-SARS-CoV-2 vaccine, we asked whether they were willing to get the second one and, if not, why.

The fifth part was about the participant’s motivation for receiving an anti-SARS-CoV-2 vaccine. The participant answered by responding to different options according to a Likert scale (1 = not at all; 7 = very much). (To what extent was the choice to get vaccinated due to: a concern to protect my health; a concern to protect the health of the community; civic sense; an employer’s request; advice from my medical doctor).

The sixth part regarded the emotion experienced by the participant after receiving an anti-SARS-CoV-2 vaccine. The emotions inquired about were happiness and worry. Happiness was defined as including experience of a highly desirable and positive emotional state understood in terms of a high-arousal state such as excitement and a sense of personal achievement [68]; worry was defined as an emotional state characterized by the experience of anxious thoughts about a potential negative event [69], one closely related to an individual’s personal goals, preferences and behaviors [70,71].

The participant answered according to a Likert scale (1 = not at all; 7 = very much).

The seventh part investigated the kind and amount of information that the participant had received before getting vaccinated. The participant answered according to a Likert scale (1 = not at all; 7 = very much). The information channels inquired about included TV, radio, pages for scientific dissemination on social media, newspaper articles shared on social media, trusted colleagues, seminars proposed by working structures and scientific publications.

In the eighth and final part, we asked participants if and how they were promoting the acceptance and diffusion of anti-SARS-CoV-2 vaccines within the community (via social networking, the scientific literature, personal experience and posing risks and benefits, or not promoting).

#### 2.3.3. Personality Trait Measurements

The third area of the survey refers to the personality traits of the participants. In our search for an instrument to measure individual differences in personality, the Ten-Item Personality Inventory (TIPI) [72] seemed to be most suitable. The widely used and validated TIPI is a short version of the Big Five personality questionnaire and for time-limited contexts or large survey questionnaires it appeared to be the most adequate instrument. The Ten-Item Personality Inventory [73] was developed using descriptors from other well-established Big Five instruments. Each item consists of two descriptors, separated by a comma, using the common stem “I see myself as:”. Each of the ten items is rated on a 7-point scale ranging from 1 (strongly disagree) to 7 (strongly agree). The ten items are: (1) extroverted, exuberant; (2) difficult, adversarial; (3) trustworthy, self-disciplined; (4) worried, anxious; (5) open to new experiences, with many interests; (6) reserved, silent; (7) understanding, affectionate; (8) disorganized, absent-minded; (9) calm, emotionally stable; (10) traditionalist, routine-bound. The result of the test is a five-factor personality structure, the factors being: agreeableness, conscientiousness, emotional stability or neuroticism, extroversion, openness.

#### 2.3.4. Plan of Analysis

The data were analyzed using the statistical software R [74]. First, descriptive statistics were computed for the original survey data and the TIPI scale data. Mean and associated SD values as well as bivariate correlations were reported for the investigated variables, namely, emotions of happiness and worry after the vaccine; personal factors, including personality characteristics, age and education; and environmental factors, including vaccination confidence, perceived risk of COVID-19 and the amount of information participants had received regarding vaccines. In addition, we performed a series of regression analyses to explore the role of personal and environmental factors as predictors of change in emotional reactions (happiness and worry) and to explore the types of variables that contributed most to responses to anti-SARS-CoV-2 vaccines. More specifically, the following models were tested and compared: (1) Model 1, including age and education as predictors of happiness and worry; (2) Model 2, concerning personality as a predictor of happiness and worry; (3) Model 3, concerning vaccination confidence as a predictor of happiness and worry; (4) Model 4, concerning vaccination confidence and the amount of vaccine information received as predictors of happiness and worry; (5) Model 5, concerning vaccination confidence, amount of vaccine information received and COVID-19 perceived risk as predictors of happiness and worry; (6) Model 6, including all variables as predictors of happiness and worry. To compare the models and select the one that received the most support, we used comparative fit indices—the Akaike Information Criterion (AIC) and the Bayesian Information Criterion (BIC)—with lower values providing more support for one model as against the previous tested model [75]. Third, for an explorative target, the correlations between emotions after vaccination (happiness and worry) and the various modalities of vaccine promotion were examined (effect size: <0.10 = trivial, <0.30 = low, >0.30 = moderate, >0.50 = strong).

## 3. Results

### 3.1. Descriptive Statistics

**Figure 1:**
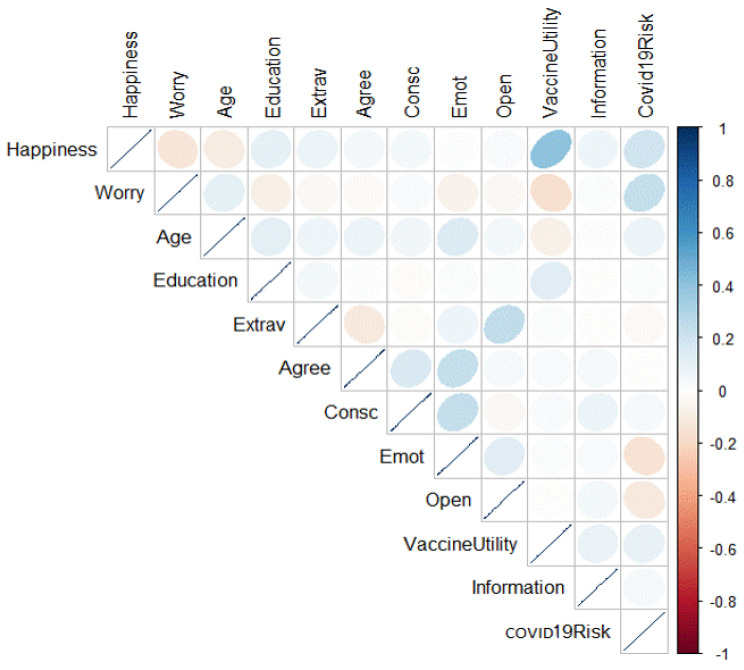
Graph of correlation matrix of the data. Correlation coefficients are colored according to value.

Overall, participants reported a high level of confidence in vaccination (M = 6.71; SD = 0.73) and medium-level risk perception of COVID-19 (M = 4.43; SD = 1.75). In addition, they judged themselves to have an above-average level of knowledge about COVID-19 (M = 5.28; SD = 1.30). Mostly, this knowledge came from the scientific literature (M = 5.44; SD = 1.79), followed by pages for scientific dissemination on social media (M = 4.88; SD = 1.91), trusted colleagues (M = 4.35; SD = 2), newspaper pages on social media (M = 3.17; SD = 1.95), TV or radio (M = 2.59; SD = 1.8) and seminars proposed by the working structure (M = 2.41; SD = 2.08). Regarding the emotions felt after vaccination, participants claimed to have felt more happy (M = 6.04; SD = 1.44) than worried (M = 2.74; SD = 1.90). In respect of how participants were promoting anti-SARS-CoV-2 vaccines within the community, the most frequently used modality was sharing personal experience with the vaccine (75.9%), followed by sharing the risks and benefits of vaccination (42%) and posting photos about the vaccination day or reposting other people’s posts about vaccination or scientific content on social media (41.9%) and sharing scientific literature (35.1%), while some stated that they were not promoting the vaccine (8.9%) (Table A2). Bivariate associations are reported in Table A3. A graph of a correlation matrix of the data is shown in Figure 1. Associations were overall low to moderate, with effect sizes ranging from 0.04 to 0.40.

### 3.2. Regression Models: Variables for Emotions

*Happiness*. The model that received most support compared to the others was the one including vaccine utility (Model 5), COVID-19 risk perception and quantity of information regarding the vaccine as predictors of happiness (adjusted R-squared = 0.188; F-statistic = 246.7; DF = (33,180); *p*-value = 0.001). AIC–BIC weights are reported in Table 1; regression parameters are reported in Table 2. Specifically, this variable was positively predicted by all three factors.

*Worry*. The model that received most support compared to the others was the one including all the variables investigated (adjusted R-squared = 0.1041; F-statistic = 34.17; DF = (102,844); *p*-value = 0.001). AIC–BIC weights are reported in Table 1; regression parameters are reported in Table 3. Specifically, this variable was positively predicted by age and perceived risk of COVID-19 and negatively by education, neuroticism and vaccination confidence.

### 3.3. Associations between Variables

To approach the question of the relations between emotions and the different modalities of vaccination promotion, a correlational analysis was performed. Table 4 shows that only “not promoting vaccination” had a negative correlation with the emotion of happiness. On the other hand, regarding the emotion of worry, promotion through social networking and narratives of personal experience correlated negatively, while not promoting vaccination correlated positively.

## 4. Discussion

The aims of this study were to show what influenced the emotions experienced by healthcare workers after being vaccinated with an anti-SARS-CoV-2 vaccine and how the emotional impact after vaccination related to modalities of promotion of vaccine uptake within the community. The pandemic had a considerable impact on the healthcare system and its professionals, leading this population to experience high levels of burnout and psychological symptoms [62]. This population was the first to be vaccinated in Italy, and research shows that people who had higher levels of trust in information from healthcare professionals were more likely to accept a vaccine [3,21,23,36]. Healthcare workers with more positive views on vaccination were also more likely to guide hesitant patients towards accepting a vaccine [36,54,64,76,77]. Hence, understanding the emotional impact of vaccination on this population is fundamental to achieving future mass vaccine coverage. In order to identify possible predictors of the emotions of happiness and worry after vaccination, we looked at the influence of various personal and environmental factors. We found that higher perception of COVID-19 risk played a role as a predictor of happiness after vaccination. This may be due to the fact that risk perception, understood in terms of concern about contracting the disease, encourages vaccination choice [78]. This attitude was heightened among healthcare providers, who had to come face to face with a new virus to a greater extent than other members of society. Together with this factor, vaccine confidence also appeared to be a predictor of happiness. In fact, having a high vaccine confidence leads to positive emotional feelings post-vaccination. This is fundamental in the population of healthcare providers, considering that they are required to talk to and sensibilize patients and laypeople to anti-SARS-CoV-2 vaccination [32,33,34,35]. The last predictor of happiness was the amount of information received before vaccination. Our data show that participants mostly received scientific information, for example, by consulting the scientific literature and scientific dissemination pages on social media [16,58]. Therefore, it is important to underline that the kind of information received about anti-SARS-CoV-2 vaccines has an impact on the emotions felt after vaccination. Indeed, this result is crucial considering the role of healthcare workers as scientific educators in these circumstances. Hence, we underline the importance of the role of healthcare workers and their responsibility to adequately inform themselves and refer to suitable sources of information in advising patients [58]. In addition, we were interested to understand whether and how healthcare workers promoted vaccine uptake. Our results show that vaccination promotion by any type of channel was associated with happier emotional status after vaccination. The use of different types of channels allows the targeting of diverse and wider audiences [16]. Indeed, we can suppose that the adoption of a more scientific attitude towards anti-SARS-CoV-2 vaccines leads to more positive feelings after vaccination which can, in turn, boost promotion of vaccine uptake. Therefore, instead of fearing the risks of a new vaccination, this population was happy to be vaccinated.

Regarding the emotion of worry after vaccination, our study shows that the personal predictors were the age of the participant, their personality traits and their level of education. The environmental predictors were the participant’s perceived risk of COVID-19, their vaccine confidence and the quantity of information they had received before being vaccinated. Older participants were more worried post-vaccination, perhaps due to possible side effects which could be more severe in older adults [29,79]. Another personal factor that can have an impact on the feeling of worry after vaccination is neuroticism. Indeed, personality traits have an impact only on the emotion of worry, supporting the strength of COVID-related predictors of emotional status. The literature shows that negative feelings have been consistently linked to neuroticism [26,80]. Furthermore, a lower level of education is associated with a greater feeling of worry post-vaccination. Lower education can lead to the indiscriminate use of information—a failure to rely on critical thinking which can lead to less considered choices [20,22]. Indeed, vaccine confidence decreases with educational level, which was revealed to be a predictor of worry after vaccination [20,33]. This predictive factor is fundamental in the case of healthcare workers because of their role in the vaccine sensibilization and trust campaign [58]. Lastly, a higher perception of COVID-19 risk is associated with greater worry. This may be due to a general emotional activation caused by the COVID-19 pandemic and related side effects.

These findings should be interpreted in light of some limitations to the study. First, the data were collected via the internet, so they may have been influenced by selection bias and may have also excluded older people. On the other hand, the survey company that facilitated the data collection (Qualtrics, Provo, UT, USA) provide a means of gaining quick access to a large and diverse sample, especially during pandemic conditions. Second, the data were collected during the first months of vaccination; thus, the results will have been affected by these social circumstances. However, we were interested in the jumpstart phase of vaccination, which occurred in the first months of 2021. Third, questions regarding the vaccine were based on the literature known at the time of the survey distribution. Continued monitoring throughout the pandemic and throughout the development of anti-SARS-CoV-2 vaccines will help us to understand the changing outcomes in terms of feelings. Fourth, we performed a short pilot study in order to test intelligibility, but a larger study was necessary in order to validate the instrument. We did, however, have a high completion rate, a representative sample and, most importantly, this was the first study that looked at a vaccinated population.

Our population was composed of healthcare workers who deal with COVID-19 patients and who were the first to receive a COVID-19 vaccine. With respect to the modalities of vaccine promotion, this factor may be associated with a happier emotional status after vaccination, and we can speculate that people who were more worried after vaccination tended to be less willing to promote vaccine uptake. We can deduce that the results observed were the product of a complex interaction between personal, social and environmental factors which could have different effects on vaccinated individuals’ emotions and, in turn, on the vaccination campaign. The novelty of the COVID-19 virus and anti-SARS-CoV-2 vaccines led to new psychological side effects largely due to widespread misinformation in the media and the misreporting of certain vaccinated individuals who represented the first source of information for the rest of the public. Our study shows that there is an apparent necessity to improve and increase health literacy regarding COVID-19 vaccines among healthcare workers by various educational means and to underline the fundamental role they play in the vaccination campaign which has herd immunity as its aim. In fact, through the enhancement of health literacy regarding vaccination, greater compliance in regard to vaccination promotion and more competent communication to the general population can be expected. Indeed, it is essential to improve institutional and scientific communication among healthcare workers in order to enhance their roles as vaccination promoters and facilitators. Furthermore, it is important to boost their participation in the promotion process, focusing more on positive than on negative messages in order to improve collaboration among the whole community.

## 5. Conclusions

The pandemic caused by the spread of COVID-19 led to great psychological distress among the entire world population but especially among healthcare workers. Healthcare workers were the first to receive an anti-SARS-CoV-2 vaccine. Understanding the predictors of psychological side effects within this population is fundamental in order to promote vaccination and achieve herd immunity, especially considering the role that healthcare workers still have to play in the vaccine sensibilization campaign. Our results showed that the emotional impact of vaccination on healthcare workers emerged from a complex interaction between personal, social and environmental factors, underlining the important role of a scientific attitude and health literacy in producing positive emotions. Furthermore, vaccination promotion is associated with happier emotional status after vaccination. The use of these findings can support preventive approaches, while potentially improving the anti-SARS-CoV-2 vaccine campaign. This study may help to identify the needs of healthcare workers in relation to this campaign and aid in the design of new methods and procedures to best promote vaccine acceptance through a scientific approach. This is even more important because most vaccine platforms use a two-dose prime boost approach to generate an immune response [81]. Furthermore, vaccination programs must maximize early impact, particularly with the accelerated spread of new variants. Indeed, it is necessary to increase healthcare workers’ health literacy in relation to anti-SARS-CoV-2 vaccines, promote advertising campaigns based on scientific knowledge and refute falsehoods about vaccine safety and efficacy. Future research should thoroughly study the psychological side effects of vaccination throughout the entire vaccination campaign, compare populations of different cultures, enlarge the sample size and look at other variables that could have an impact on vaccination promotion by healthcare workers within their communities.

## Figures and Tables

**Table 1:** AIC–BIC weights for the compared models.

	Happiness		Worry	
	AIC	BIC	AIC	BIC
Model 1	20,047.18	20,073.74	22,591.11	22,617.56
Model 2	18,831.12	18,877.19	21,293.84	21,339.74
Model 3	10,990.02	11,008.35	13,374.31	13,392.55
Model 4	10,397.43	10,421.7	12,793.261	12,817.41
Model 5	10,299.23	10,329.56	12,543.95	12,574.13
Model 6	9191.746	9263.494	11,434.22	11,505.7

**Table 2:** Regression analysis: variables for happiness.

Model 5	B	t	*p*
Intercept	0.00000	1.66	0.095
Vaccine utility	0.36464	22.58	0.001 ***
COVID-19 risk	0.15001	9.30	0.001 ***
Information	0.10252	6.33	0.001 ***

Note: *** *p* < 0.001.

**Table 3:** Regression analysis: variables for worry.

Model 6	B	t	*p*
Intercept	0.000	11.50	0.001 ***
Age	0.07165	3.81	0.001 ***
Education	−0.07716	−4.19	0.001 ***
Extraversion	−0.02631	−1.41	0.157
Agreeableness	−0.01991	−1.07	0.284
Consciousness	0.03002	1.63	0.102
Emotional stability	−0.0623	−2.25	0.0241 *
Openness	−0.0251	−0.72	0.470
Vaccine utility	−0.1672	−9.26	0.001 ***
COVID-19 risk	0.25152	13.76	0.001 ***
Information	0.01366	0.74	0.455

Note: * *p* > 0.01 and < 0.05; *** *p* < 0.001.

**Table 4:** Correlational analysis. Different modalities of vaccine promotion and emotions of happiness and worry after vaccination.

	Happiness	Worry
Social network	0.21 **	−0.45 **
Scientific literature	0.15 **	−0.02
Personal experience	0.21 **	−0.06 **
Risks and benefits	0.06 **	−0.01
Not promoting	−0.25 **	0.06 **

Note: ** *p* < 0.01 and > 0.001.

## Appendix A

**Table A1:** Sample qualification.

Qualification	N	%
Medical doctors	3211	55.5
Dentists	111	1.9
Nurses	1549	26.8
Other healthcare figures	491	8.5
Operator social health (OSS)	70	1.2
Pharmacists	43	0.7
First aid workers	34	0.6
Medicine students	71	1.2
Nurses students	32	0.6
Administratives	42	0.7
Others	132	2.3

**Table A2:** Modalities of promotion of vaccine acceptance.

Modalities	N	%
Social networking	2427	41.9
Scientific literature	2035	35.1
Personal experience	4397	75.9
Sharing risks and benefit	2429	42
Not promoting	513	8.9

**Table A3:** Descriptive statistics of study variables: means, associated standard deviations and Pearson’s r bivariate correlations.

	M (SD)	1	2	3	4	5	6	7	8	9	10	11
1. Age	1.80 (0.76)											
2. Education	5.09 (0.93)	0.11 **										
3. Extraversion	4.16 (1.39)	0.06 **	0.04 **									
4. Agreeableness	5.43 (1.02)	0.07 **	0.008	−0.118 **								
5.Conscientiousness	5.55 (1.07)	0.05 **	−0.017	−0.012	0.158 **							
6. Emotional stability	4.68 (1.32)	0.14 **	0.013	0.063 **	0.231 **	0.231 **						
7. Openness	4.50 (1.02)	0.04 **	0.016	0.249 **	0.032 *	−0.032	0.123 **					
8. Vaccine utility	6.71 (0.73)	−0.07 **	0.129 **	0.11	0.027	0.029	0.017	−0.005				
9. Covid-19 risk	4.43 (1.75)	0.07 **	0.019	−0.021	−0.002	0.036 **	−0.143 **	−0.118 **	0.98 **			
10. Information	5.28 (1.30)	0.13 **	0.038 **	0.051 **	0.040 **	0.101 **	−095 **	0.085 **	0.137 **	0.160 **		
11. Happiness	6.04 (1.44)	−0.09 **	0.107 **	0.077 **	0.047 **	0.040 **	0.004	0.025	0.403 **	0.193 **	0.177 **	
12. Worry	2.74 (1.90)	0.100 **	−0.082 **	−0.038 **	−0.024	0.029 *	−0.067 **	−0.039 **	−0.161 **	0.238 **	0.014	−0.140 **

Note: * *p* > 0.01 and < 0.05; ** *p* < 0.01 and > 0.001.
